# Atypical presentation of primary intraocular lymphoma

**DOI:** 10.1186/s12886-016-0350-x

**Published:** 2016-10-03

**Authors:** Koji Komatsu, Tsutomu Sakai, Toshikatsu Kaburaki, Hideki Tsuji, Hiroshi Tsuneoka

**Affiliations:** 1 Department of Ophthalmology, Jikei University School of Medicine, 3-25-8 Nishishimbashi, Minato-ku, Tokyo, 105-8461 Japan; 2 Department of Ophthalmology, The University of Tokyo Graduate School of Medicine, Tokyo, Japan; 3 Department of Ophthalmology, The Cancer Institute Hospital of Japanese Foundation for Cancer Research, Tokyo, Japan

**Keywords:** Primary intraocular lymphoma, Vitelliform submaculopathy, Spectral-domain optic coherence tomography, Case report

## Abstract

**Background:**

In 2014, Pang et al. reported three cases with vitelliform submaculopathy as a preceding lesion of primary intraocular lymphoma (PIOL). Here, we report a case with an atypical presentation of PIOL who initially presented with vitelliform submaculopathy, vitreous haze and preripheral retinal focus.

**Case presentation:**

A 73-year-old female initially visited another hospital with a chief complaint of acute reduced vision in the right eye. Funduscopic examination of the right eye showed a yellowish retinal lesion at the fovea with vitreous haze and retinal foci scattered in the peripheral region. Spectral-domain optic coherence tomography (SD-OCT) revealed a hyperreflective subretinal debris above the retinal pigment epithelium (RPE) at the fovea, suggesting vitelliform submaculopathy. Vitrectomy was performed to improve visualization of the retinal lesions and for examination of PIOL. Vitreous cytology was class III and cytokine analysis of vitreous fluid showed increased IL-10 and an IL-10/IL-6 ratio >1, suggesting PIOL. Thereafter, there was a sub-RPE infiltration of presumed lymphoma in the nasal retina, and PCR analysis of anterior chamber fluid indicated IgH gene rearrangement, leading to diagnosis of PIOL. Three months later, there was complete disappearance of the vitelliform submacular lesion, with resultant disruption and thinning of the outer retinal layers on SD-OCT images.

**Conclusions:**

Clinicians should be aware of atypical manifestations of PIOL such as vitelliform submaculopathy and peripheral retinal foci with vitreous haze. The patient’s unusual funduscopic changes are findings that have not reported in patients with PIOL.

## Background

Intraocular lymphomas are a rare form of ocular malignancy and involve two types. Primary intraocular lymphoma (PIOL) affects the vitreous, retina, choroid or optic nerve and secondary intraocular lymphoma is seen in patients who have systemic lymphoma [1]. This disease has a poor prognosis with a 5-year survival rate of 61 %, and the incidence has increased recently [2]. The disease is typical of masquerade syndromes and is often difficult to diagnose if no characteristic vitreous haze or subretinal focus is present. In 2014, Pang et al. described three cases in which vitelliform submaculopathy preceded typical lesions of PIOL, as the first report of this event [3]. Foci in those cases spontaneously disappeared in a brief period, with a subsequently diagnosis of PIOL. Here, we describe an atypical presentation of PIOL in a patient who initially presented with vitelliform submaculopathy, vitreous haze and preripheral retinal focus, in which PIOL was diagnosed in the subsequent course.

## Case presentation

The patient was a 73-year-old Japanese woman who had been suffering from visual loss and a history of floaters in the right eye from May 2012. The right eye had vitreous haze and retinal white foci in the peripheral fundus, the causes of which were unclear in a detailed examination. For further examination and treatment, she was referred to Jikei University Hospital and visited in June 2012. She was immunocompetent and had no underlying systemic diseases.

**Fig. 1:**
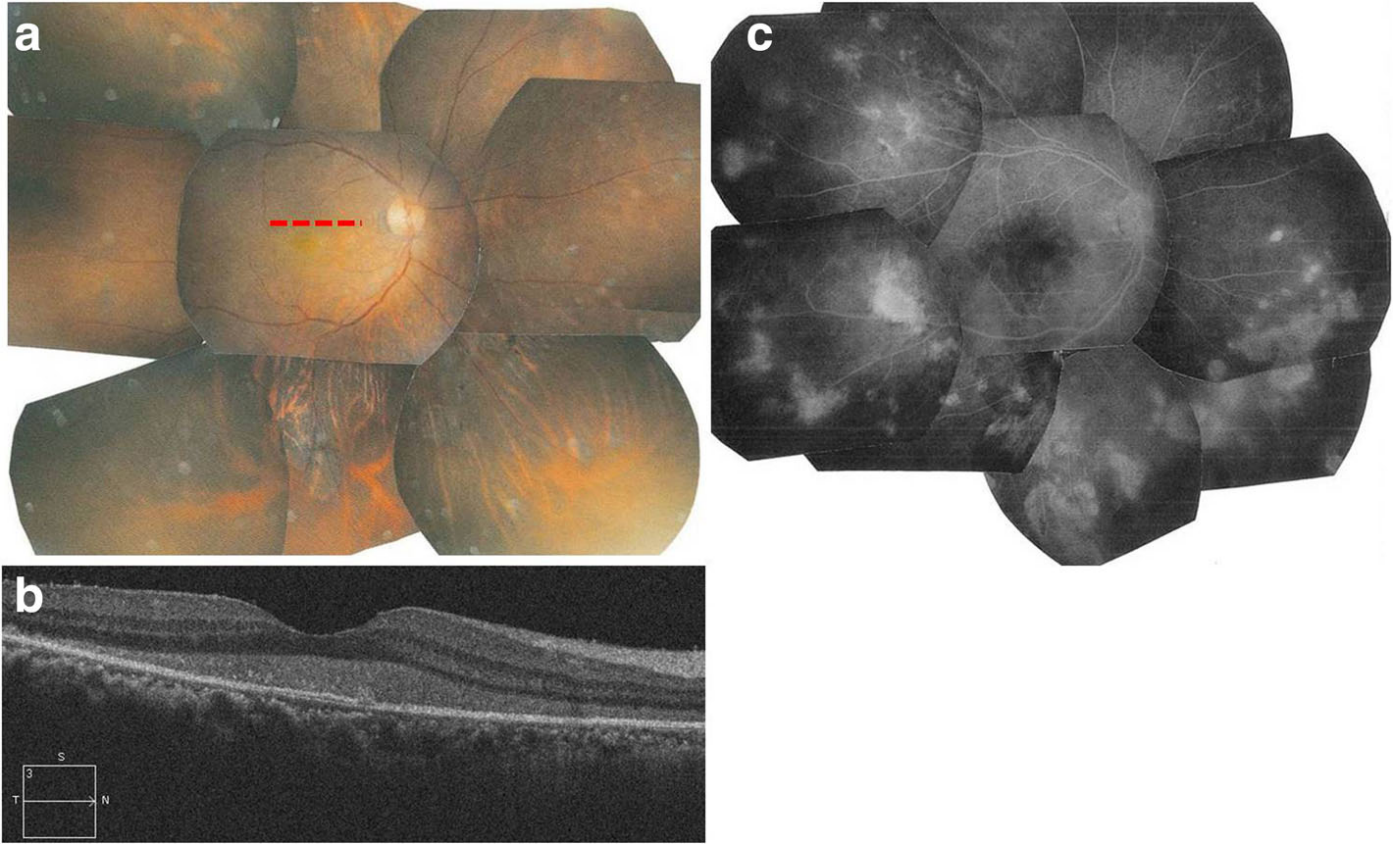
Fundus photograph, fluorescein angiography and spectral-domain optical coherence tomography (SD-OCT) at initial presentation. **a**. Fundus photograph showing submacular yellow material at the fovea and multiple peripheral retinal foci. **b**. Fluorescein angiography showing multiple hyperfluorescent peripheral lesions. **c**. SD-OCT revealed hyperreflective material deposition above the retinal pigment epithelium (RPE) band consistent with submacular yellow material

On presentation, the best-corrected visual acuity (BCVA) was 0.02 OD and 1.2 OS. There was no relative afferent pupillary defect. The patient had no cells in the anterior chamber and diffuse vitreous cells with trace haze in the right eye. Funduscopic examination of the right eye showed a yellow submacular lesion at the fovea, in addition to vitreous haze and retinal foci scattered in the peripheral region (Fig. 1a). The left eye appeared normal. Spectral-domain optic coherence tomography (SD-OCT) revealed hyperreflective subretinal debris resembling vitelliform deposition above the retinal pigment epithelium (RPE) band (Fig. 1b). Fluorescein angiography (FA) imaging showed multiple hyperfluorescent lesions of various sizes in the peripheral region (Fig. 1c). Serologic test results, including those for antitoxoplasma IgG and IgM antibodies, angiotensin-converting enzyme, and viral antibodies such as herpes simplex virus, varicella zoster virus and cytomegalovirus, were within normal limits. Chest X-ray and magnetic resonance imaging were unremarkable.

**Fig. 2:**
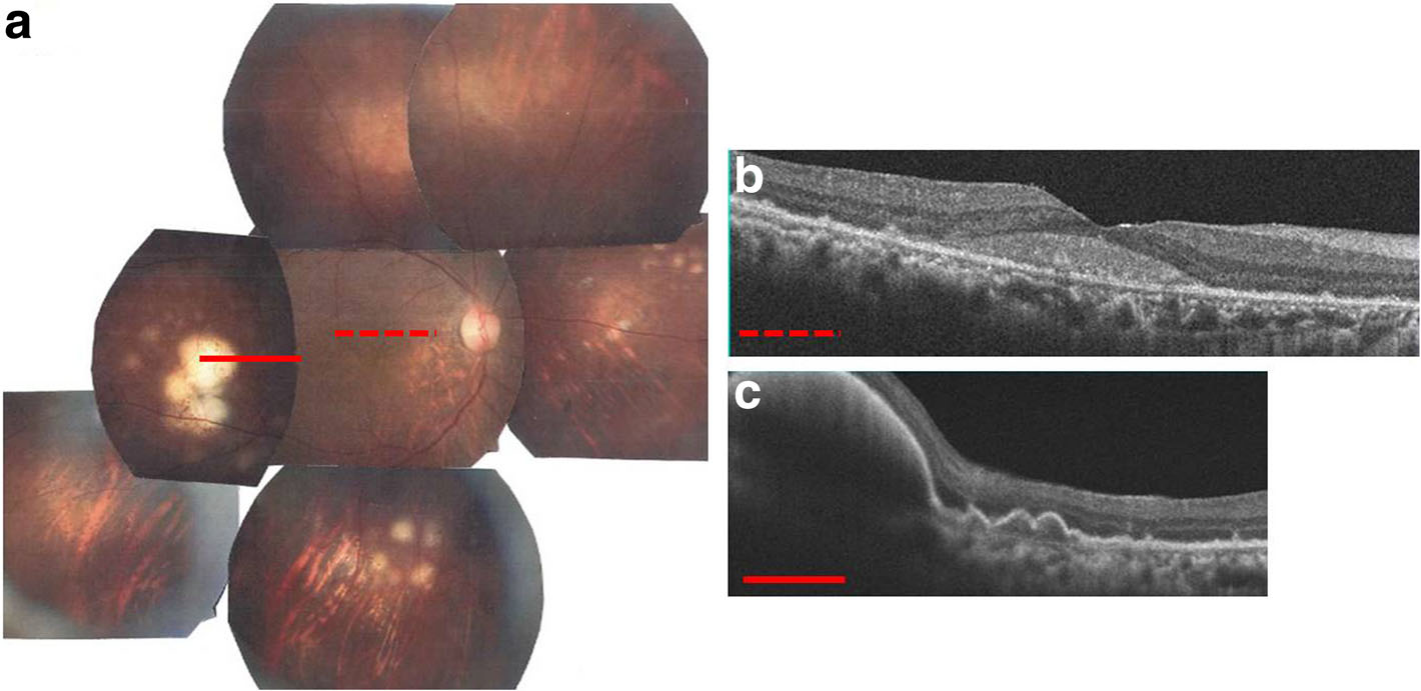
Fundus photograph and spectral-domain optical coherence tomography (SD-OCT) at 3 months after initial visit. **a**. Fundus photograph showing sub-retinal pigment epithelium (RPE) white mass in the temporal region. **b**. There was decrease in the vitelliform debris at the fovea seen with SD-OCT. **c**. SD-OCT revealed steep elevation of the RPE band consistent with sub-RPE mass

**Fig. 3:**
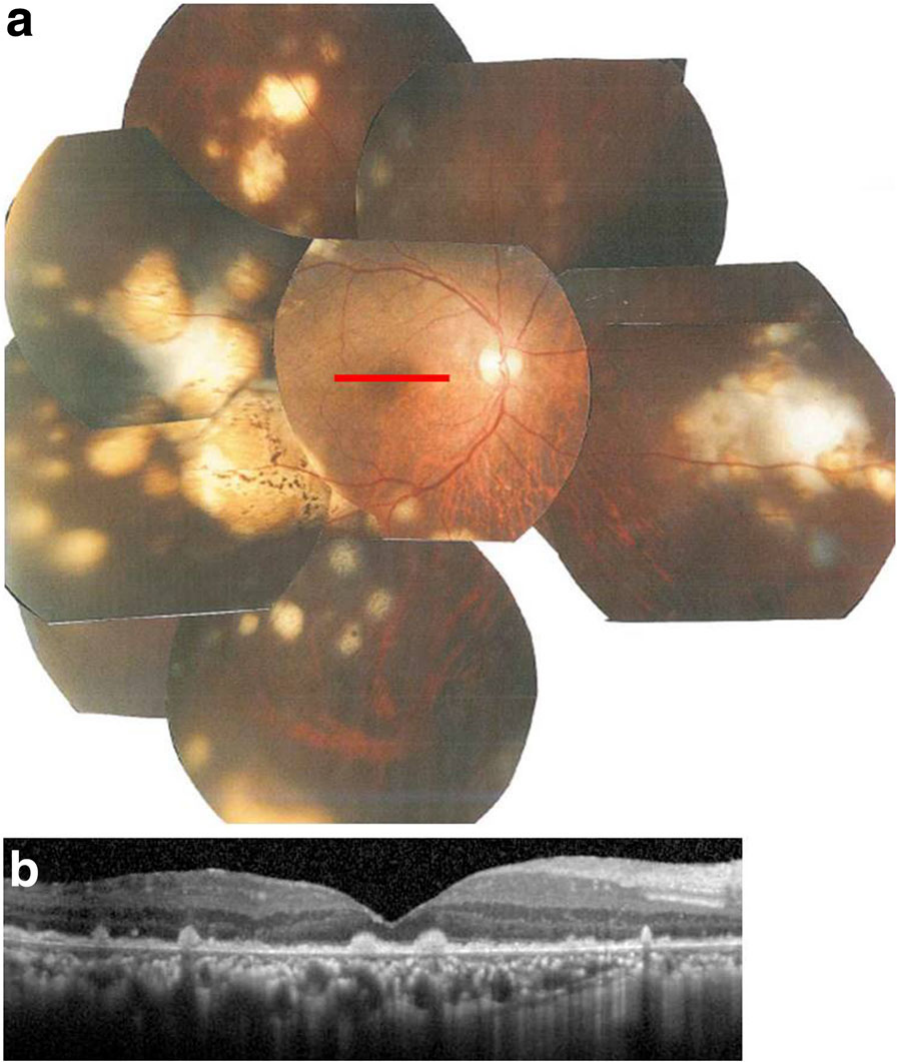
Fundus photograph and spectral-domain optical coherence tomography (SD-OCT) at 6 months after initial visit. **a**. Fundus photograph showing multiple sub-retinal pigment epithelium (RPE) white mass in the temporal and nasal region. **b**. There was decrease in the vitelliform debris at the fovea seen with SD-OCT. C. SD-OCT revealed complete resolution of the vitelliform debris and hyperreflective nodules at the RPE level

Vitreous haze slowly worsened within a few months. A 25-gauge pars plana vitrectomy was performed to improve visualization of the retinal lesions and for examination of causative microorganisms or PIOL. Vitreous cytology was class III and the cytokine analysis of vitreous fluid revealed increased IL-10 and an IL-10/IL-6 ratio >1, suggesting PIOL. Three months later, there were appearances of new multiple yellow-white sub-RPE infltrates in the peripheral fundus (Fig. 2a, c). At this time, SD-OCT still showed a hyperreflective material above the RPE band (Fig. 2b). PCR analysis of the anterior chamber fluid revealed IgH gene rearrangement, thus allowing a definitive diagnosis of PIOL. In a few months later, SD-OCT revealed hyperreflective bands and nodules above the RPE band with regression of the vitelliform debris (Fig. 3). Finally, the patient decided to treat with intravitreal methotrexate injections (weekly 400 μg/100 mL for 6 weeks). The patient has survived with a total-follow up of 31 months, with no invlolvement of the central nervous system. The right eye has remained recurrence-free 24 months after the 20th intravitreal methotrexate injections. BCVA improved to 0.4 in the right eye.

## Discussion

This case highlights some atypical features that can be associated with PIOL. On initial presentation, vitreous haze, vitelliform submaculopathy and peripheral retinal foci were present simultaneously, with subsequent appearance of sub-RPE infiltrates, a typical lesion of PIOL. This was followed by multiple lesion occurrence and growth, together with spontaneous disappearance of vitelliform submaculopathy.

In 2014, Pang et al. reported three cases in which vitelliform submaculopathy preceded typical lesions of PIOL [3]. Our case has several different features in clinical course, unlike their cases. First, our case presented with vitreous haze, vitelliform submaculopathy and peripheral retinal foci as an initial presentation, in contrast to Pang et al. cases. Secondly, in our case, there was development of sub-RPE infiltration in the peripheral retina without regression of vitelliform submaculopathy. Although Pang et al. pointed out the importance of vitelliform submaculopathy as a preceding lesion in PIOL, we thus think this condition may be involved in one of initial manifestations of PIOL. Finally, there was an occurrence of hyperreflective nodules at the RPE level and disruption of the ellipsoid zone after complete disappearance of vitelliform submaculopathy. In 13 cases of PIOL described by Keino et al., nearly one-half of the cases involved hyperreflective nodules at the RPE level and disruption of the ellipsoid zone during follow-up [4]. Although Keino et al. did not investigate an association SD-OCT patterns with clinical course, we suggest that the occurrence of vitelliform submaculopathy can be an early form of lymphoma-associated retinopathy and this condition may be seen with various manifestations in the initial phase of PIOL.

## Conclusions

PIOL can present with atypical fundus manifestations such as vitelliform submaculopathy and peripheral retinal foci with vitreous haze. SD-OCT may be useful for early detection and monitoring of small macular abnormalities in this disease.

## Abbreviations

BCVA: Best-corrected visual acuity
PIOL: Primary intraocular lymphoma
RPE: Retinal pigment epithelium
SD-OCT: Spectral-domain optic coherence tomography.
